# A Case Report of Sheehan Syndrome: A Rare Cause of Hypopituitarism

**DOI:** 10.7759/cureus.53544

**Published:** 2024-02-04

**Authors:** Ana Luís Vasconcelos, Rita Pinto Ribeiro, Patrícia Claúdio Ferreira, Joana Maciel, Rosário Araújo

**Affiliations:** 1 Internal Medicine, Hospital de Braga, Braga, PRT; 2 Diabetes and Endocrinology, Hospital de Braga, Braga, PRT; 3 Endocrinology, Hospital de Braga, Braga, PRT

**Keywords:** hypogonadism, adrenal insufficiency, hypothyroidism, amenorrhea, postpartum hemorrhage, hypopituitarism

## Abstract

Sheehan syndrome is a rare cause of hypopituitarism characterized by pituitary gland necrosis after postpartum hemorrhage. It is a pertinent cause of maternal morbidity and mortality in developing countries with deficient obstetrical care but is frequently overlooked in developed countries where its occurrence is uncommon. We present the case of a 66-year-old female diagnosed with Sheehan syndrome more than 30 years after her last delivery complicated by postpartum hemorrhage. Although the patient presented several symptoms and signs of pituitary hormonal deficiencies, a diagnosis had never been established before. The complete laboratory analysis revealed a deficiency in the pituitary axis, and the cranial imaging showed an empty sella turcica. Hormonal replacement therapy resolved several impairments in terms of general energy, physical capacity, temperature regulation, skin characteristics, and sexual function. It also improved her cardiovascular and osteoporosis risk, and, most importantly, prevented a potential life-threatening adrenal crisis.

This report highlights the subtle manifestations of Sheehan syndrome to help clinicians establish a prompt diagnosis. Even in developed countries such as Portugal, this condition should be regarded as a potential cause of hypopituitarism.

## Introduction

Hypopituitarism results from a complete or partial deficiency in pituitary hormones [1]. Sheehan syndrome is a rare cause of hypopituitarism characterized by pituitary gland necrosis after postpartum hemorrhage [2]. In developing countries, due to deficient obstetrical care, it is still a relevant cause of maternal morbidity and mortality. However, few epidemiological studies on the condition have been performed in developed countries, but a recent retrospective analysis from Iceland reported an estimated prevalence of 5.1 per 100,000 women [3]. The obstetrical history of these patients often reveals a history of blood transfusions, hysterectomy, and admission to ICU following a massive postpartum hemorrhage [4]. There is no correlation between the degree of hemorrhage and the severity of clinical expression [5].

A retrospective study involving 114 patients revealed a diagnostic delay of 19.7 years, which could be attributed to the fact that a high proportion of women with this condition present nonspecific complaints [6]. Sheehan syndrome results in secondary adrenal insufficiency, which causes a selective deficiency in adrenocorticotropic hormone (ACTH), while the renin-angiotensin-aldosterone axis remains intact [7]. In Sheehan syndrome, clinically evident arginine-vasopressin deficiency is uncommon, but according to one study, about 30% of patients appeared to have partial arginine-vasopressin deficiency after a water deprivation test [8].

Women with this condition are frequently asymptomatic or have nonspecific symptoms until a stressful event occurs and unveils the clinical picture [4]. Approximately 55% of patients have panhypopituitarism. In cases of partial hypopituitarism, the most commonly affected hormones are growth hormone (GH) and gonadotropins, follicle-stimulating hormone (FSH), and luteinizing hormone (LH), followed by thyroid stimulating hormone (TSH), ACTH, and prolactin [6]. In a retrospective study of 60 patients with Sheehan syndrome, the most commonly reported symptoms were asthenia and adynamia in 85%, failure to resume menses in 73%, loss of axillary and pubic hair in 67%, and failure to breastfeed postpartum in 65% of patients. On physical examination, decreased or absent pubic or axillary hair was reported in 93%, dry skin in 82%, and pallor in 70%, followed by vaginal atrophy, slow reflexes, mammary gland atrophy, cognitive changes, and myxedema [9].

MRI is the most sensitive method to evaluate the hypothalamus-pituitary region. An empty sella turcica of normal size has been reported as a characteristic finding in patients with Sheehan syndrome [10]. As hypopituitarism is often not readily apparent, screening of appropriate patients is important to prevent long-term morbidity [7]. Although it is rare in developed countries, recent studies suggest that its prevalence is underestimated [2].

## Case presentation

A 66-year-old woman with a past medical history of osteoporosis and dyslipidemia was admitted to the internal medicine ward for severe acute respiratory syndrome coronavirus 2 (SARS-CoV-2) pneumoniae with hypoxemic respiratory failure. She was started on a 6 mg daily dose of dexamethasone and administered a supplemental 35% fraction of inspired oxygen. Her clinical evolution was positive and five days later she was breathing room air.

**Figure 1:**
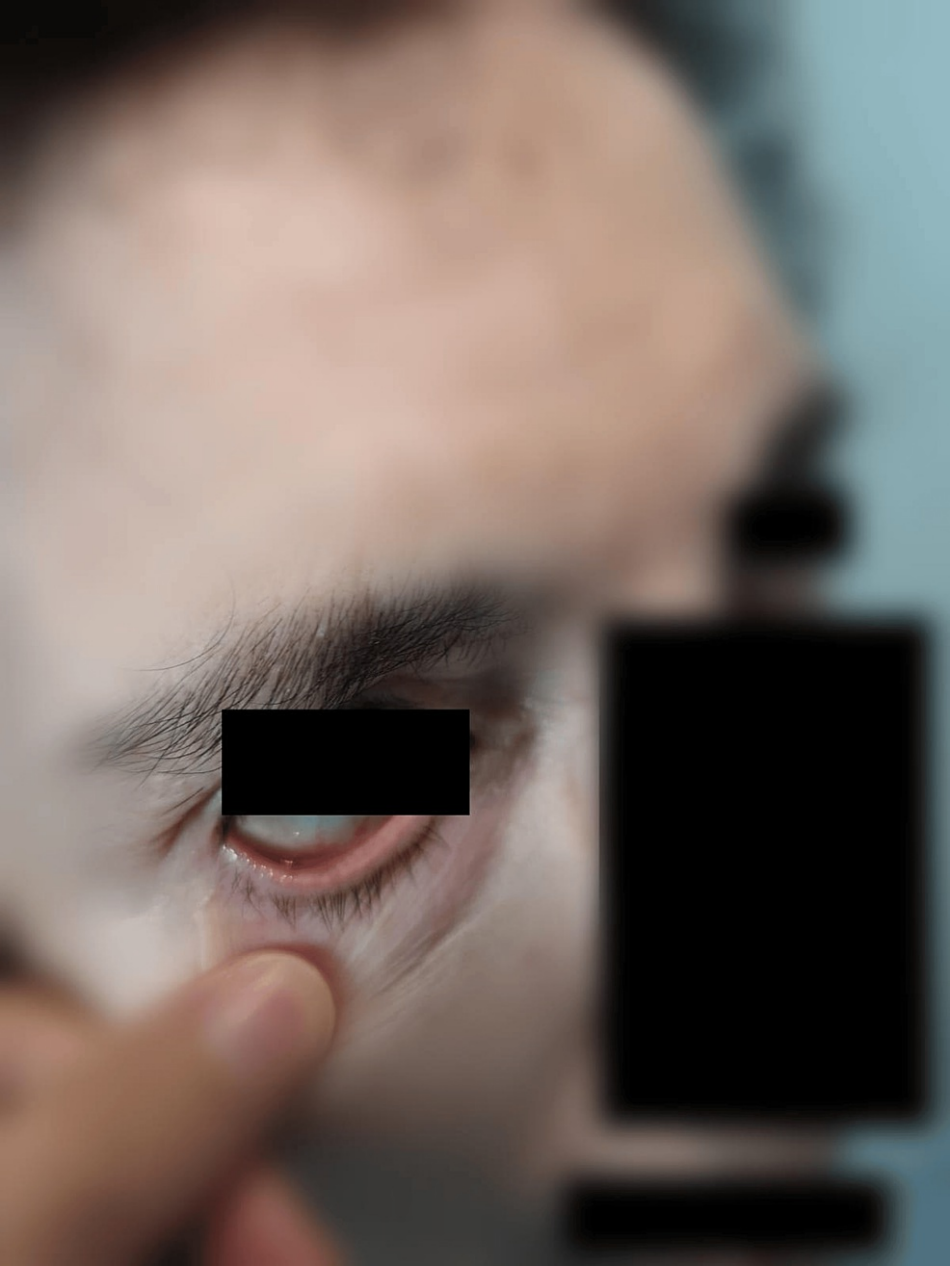
Picture of the patient’s face The picture of the patient’s face shows pale skin but a reddish eye mucosa. A marked wrinkling around the eyes and forehead is evident. Dry and weak hair is also partially seen. These findings raised concerns about cortisol and thyroid hormone deficiencies

**Figure 2:**
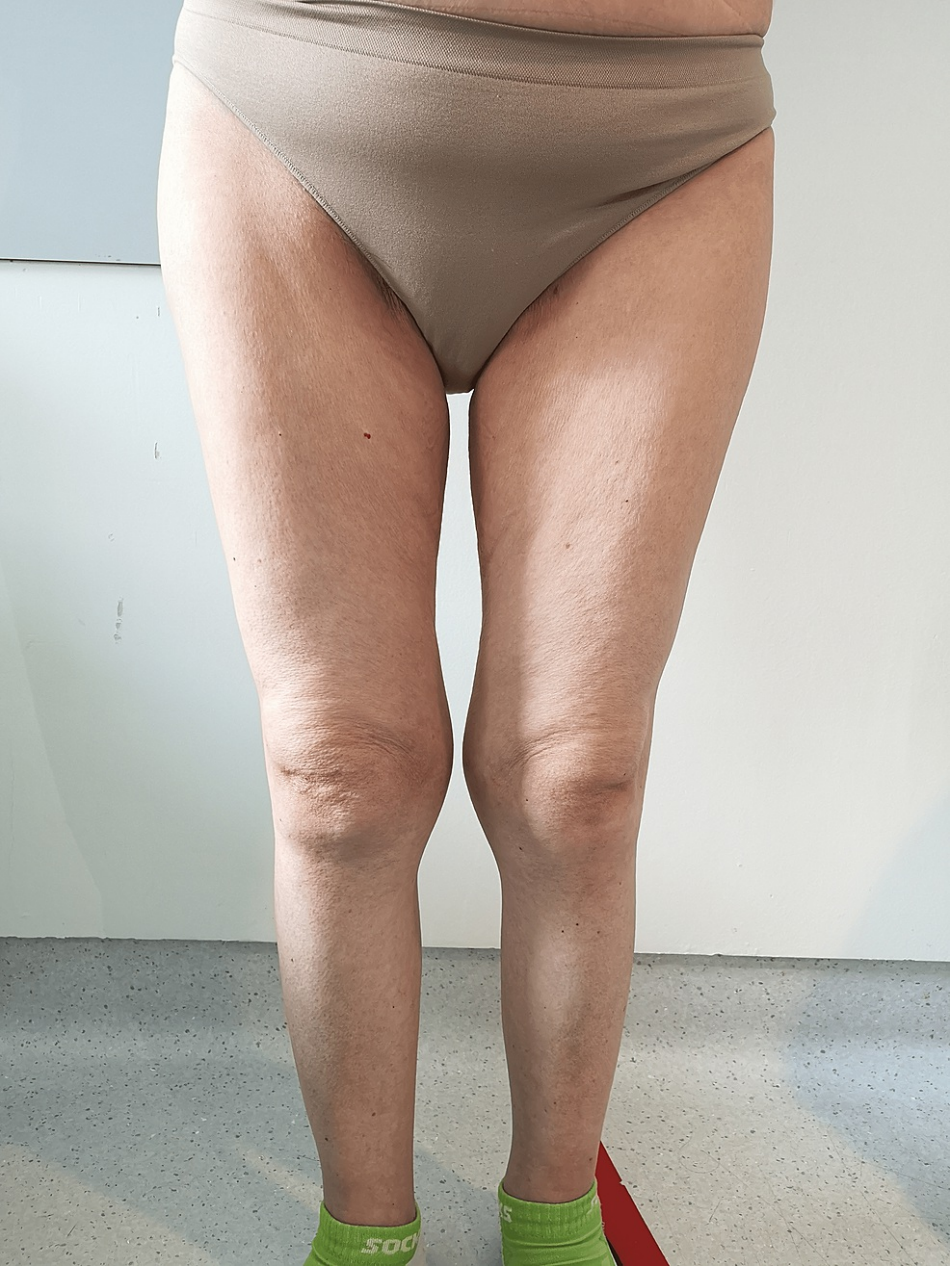
Picture of the patient’s legs The picture of the patient’s legs shows the complete absence of hair. This finding suggested that androgen deficiency due to secondary adrenal insufficiency, as well as hypothyroidism and hypogonadism, could be present

During the hospital stay, her physical examination raised some concerns. The patient exhibited generalized skin paleness without mucosal discoloration, suggesting true hypopigmentation instead of anemia (Figure 1). Her skin appeared dry; she had marked wrinkling around the mouth and the eyes and her hair was thin. A decrease in pubic hair and complete absence of axillary or remaining body hair were also noticed (Figure 2). Her movements were extremely slow but muscle strength was preserved in all segments. A gynecological examination revealed vaginal dryness and atrophy. Her BMI was 21 kg/m^2^. The neurological exam was normal. Her blood pressure, cardiac frequency, and body temperature were also normal.

The patient reported a long-standing history of asthenia, particularly when physical efforts were required. She had worked as an office assistant and did not recall any difficulty performing her job because she had always been seated. Her weight had been stable for many years. She felt frequently cold, even during summer, and never got tanned. She mentioned hot flashes, vaginal dryness, and dyspareunia, which had occurred many years ago. Her bowel movements were regular.

Her obstetric history of four gestations included two miscarriages and two deliveries with two live births. Her last delivery had been in 1990 at 34 years of age. She had been assisted in the hospital and a vaginal delivery had been performed, but the postpartum period had been complicated by vaginal bleeding. She had been hospitalized for one month. She recalled experiencing intense headaches and asthenia at that time. She had never been able to lactate and had become amenorrheic since then. Her laboratory analysis from two years prior revealed low levels of free thyroxine (T4) and a TSH value in the reference range. Anti-peroxidase antibodies were negative and a thyroid ultrasound was normal. The patient had never been put on thyroid hormonal supplementation.

**Figure 3:**
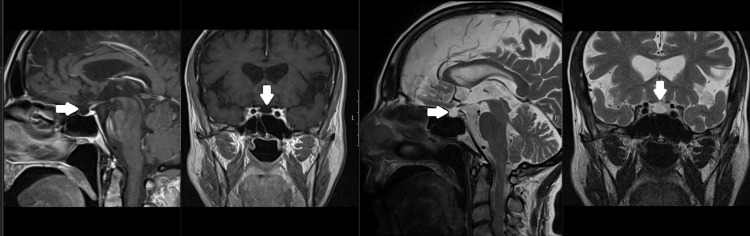
Cranial MRI images Cranial MRI of the patient showing an empty sella turcica (arrows) with pituitary tissue compressed against the sellar floor with lateral stalk deviation MRI: magnetic resonance imaging

A new panel of laboratory tests showed low levels of free T4 and free triiodothyronine (T3) and a normal TSH, suggesting central hypothyroidism. FSH and LH levels were both reduced, which was compatible with central hypogonadism. The adrenal axis was not evaluated because the patient was on dexamethasone therapy for SARS-CoV-2 pneumoniae. The 24-hour urinary volume was lower than 3L and serum sodium level was normal. A cranial MRI was performed, which showed a sella turcica of normal size filled predominantly with liquor and a pituitary parenchyma compressed against the sellar floor with a median stalk posteriorly deviated (Figure 3).

The patient's obstetrical history, symptoms, physical examination, laboratory tests, and radiology study were suggestive of hypopituitarism secondary to pituitary ischemia in the postpartum, a condition known as Sheehan syndrome. Levothyroxine supplementation at a dose of 1.6 mcg/kg was started. She was already on dexamethasone, which prevented an adrenal crisis. After suspension, she was switched to oral hydrocortisone, with a gradual tapering until 10 mg in the morning, 5 mg at lunch, and 5 mg in the late afternoon. The patient was subsequently discharged home.

Four months after discharge, the patient was clinically reevaluated as part of an internal medicine and endocrinology consultation. After initiating hydrocortisone and levothyroxine, she reported having progressively more energy, strength, faster thinking, and a need for fewer hours of sleep. Her weight was stable and she registered normal values of arterial blood pressure at her weekly check-up. At that time, we counseled her about stress-dose and emergency corticoid administration. Human growth hormone replacement was not started following a discussion about the advantages and disadvantages of this therapy with the patient.

Pituitary hormonal analysis was repeated two months later. The hydrocortisone was suspended 18 hours before the blood analysis. The patient's cortisol serum level was 4.49 ug/dL, and hence a cosyntropin stimulation test was performed, which confirmed secondary adrenal insufficiency (cortisol level of 8.21 ug/dL after 60 minutes). Thyroid function was normalized with daily 50 mcg of levothyroxine. Her FSH and LH were suppressed, age-adjusted insulin-like growth factor 1 (IGF-1) was low, and prolactin level was normal-low. These results were compatible with panhypopituitarism (Table 1).

**Table 1:** Laboratory results of pituitary hormone and other relevant tests *Blood samples were drawn 18 hours after the suspension of hydrocortisone therapy. **On levothyroxine 0.05 mg/day. ***Reference values of our hospital pathology laboratory. Age-adjusted reference values [11] are 58.95-193.70 ng/mL ACTH: adrenocorticotropic hormone; T4: thyroxine; T3: triiodothyronine; TSH: thyroid-stimulating hormone; FSH: follicle-stimulating hormone; LH: luteinizing hormone; IGF-1: insulin-like growth factor 1

Laboratory test	Before hospital admission	During hospital admission	After 6 months of hospital admission*	Reference values (unit)
Hypothalamic-pituitary-adrenal axis				
Cortisol	-	-	4.49	4.3–22.4 (ug/dL)
ACTH	-	-	20.80	<46 (pg/mL)
Cosyntropin test	-	-	Cortisol after 30’: 6.25 Cortisol after 60’: 8.21	<14 (ug/dL)
Hypothalamic-pituitary-thyroid axis				
Free T4	0.30	0.36	1.19**	0.89–1.76 (ng/dL)
Free T3	-	0.66	2.48**	2.3–4.2 (pg/mL)
TSH	3.37	1.33	0.91**	0.55–4.78 (uUI/mL)
Anti-peroxidase antibodies	Negative	-	-	-
Hypothalamic-pituitary-gonadal axis				
FSH	-	4.30	5.59	23.0–116.3 in postmenopausal women (mUI/mL)
LH	-	1.08	1.80	15.9–54.0 in postmenopausal women (mUI/mL)
Hypothalamic-pituitary-somatotropic axis				
IGF-1	-	-	48.30	40–225 (ng/mL)***
Hypothalamic-pituitary-prolactin axis				
Prolactin	-	-	3.07	1.8–20.3 in postmenopausal women (ng/mL)

We also checked her hydroelectrolytic balance as well as lipid and glycemic profiles and subsequently ordered a bone density scan. She was referred for a gynecological appointment to manage her vaginal atrophy and sexual complaints.

## Discussion

The pathogenesis of Sheehan syndrome is not completely understood. During pregnancy, the pituitary gland normally enlarges due to hyperplasia of lactotroph cells in response to estrogen stimulation [10]. The hypervascular gland is thus particularly vulnerable to arterial pressure changes [7]. The anterior pituitary is even more susceptible to ischemia because its source of blood supply is a low-pressure dense capillary network. When severe hypotension occurs due to massive postpartum hemorrhage, ischemic necrosis of the pituitary is possible. It has been proposed that autoimmunity and coagulopathy play a role but it remains unclear [10]. The clinical presentation in these patients often manifests a combination of multiple hormonal deficits. Some symptoms or signs are attributed to one specific hormone while others involve a combination of multiple deficiencies. Low libido, hot flashes, infertility, and vaginal dryness are associated with FSH and LH, while cold intolerance is linked to TSH. On the other hand, pallor, dry skin, thinning hair, and loss of body hair are a consequence of ACTH, FSH, and LH deficiencies. Asthenia, the main symptom reported, is attributed to concomitant deficient ACTH, TSH, FSH, LH, and GH [1].

The hypothalamic-pituitary-adrenal axis should be tested at least 18-24 hours after the last hydrocortisone dose. A cortisol level below 3 ug/dL is indicative of adrenal insufficiency while that above 15 ug/dL excludes this diagnosis. When cortisol values are between 3 and 15 ug/dL, a confirmatory test is recommended, with the cosyntropin stimulation test being the most widely used. The test is performed by administering 0.25 mg of cosyntropin and assessing serum cortisol level 60 minutes later. A value equal to or higher than 18 ug/dL is considered normal [1]. Central hypothyroidism is assessed by measuring free T4 and TSH levels. A free T4 level below the reference range in conjunction with a low, normal, or mildly elevated TSH in the setting of pituitary disease usually confirms the diagnosis, unless the patient is severely ill [7]. In our case, thyroid function tests during hospital admission may have been affected by the acute illness, but the previous analysis confirmed a low free T4 of 0.30 ng/dL (reference range: 0.89-1.76 ng/dL) and a normal TSH of 3.37 uUI/mL (reference range: 0.55-4.78 uUI/mL); both results were compatible with central hypothyroidism.

In postmenopausal women, the absence of high serum FSH and LH is sufficient for a diagnosis of gonadotropic dysfunction [1]. In our case, the patient was in the postmenopausal phase and showed low levels of both FSH and LH. Additionally, she had been amenorrheic since her last delivery at 34 years of age. In patients with hypothalamic-pituitary disease with three or more hormone deficiencies, if the IGF-1 serum level is below the reference range, there is more than a 97% chance of GH deficiency and a GH stimulation test is not required [7,11]. A recent European analysis established an IGF-1 reference value of 58.95 to 193.70 ng/mL for patients in the age group between 66 and 70 years [12]. In our case, a low IGF-1 value of 48.30 ng/mL in a patient with multiple pituitary hormonal deficiencies establishes a GH deficiency.

The only clinical consequence of prolactin deficiency is the inability to lactate after delivery [7]. Some authors suggest that the TRH stimulation test is the most sensitive [9]. In our case, the patient was unable to lactate, also suggesting a prolactin deficiency. Arginine-vasopressin deficiency is a rare manifestation of Sheehan syndrome. In this case, the plasma osmolarity, serum sodium, and 24-hour urinary volume were normal. A water deprivation test was not performed. Damage to the sellar diaphragm may lead to arachnoid herniation into the sellar space, a condition radiologically described as an empty sella. When it is secondary to damage to the pituitary gland, pituitary tissue compressed against the sellar floor with lateral stalk deviation is characteristically seen [7]. In our case, the patient’s MRI described the typical aspects of an empty sella turcica; there was a diagnostic delay of 32 years in our patient, a longer period than previously reported in the literature [6,9].

Replacement therapy for hypopituitarism involves an individualized regimen and frequent dose adjustment. Glucocorticoid doses typically constitute 10-20 mg of hydrocortisone daily in divided doses, with the highest dose administered in the morning, to mimic circadian rhythm cortisol secretion. In stressful situations, a higher dose is imperative to prevent an adrenal crisis. Thyroid hormone supplementation should maintain a free T4 level close to the upper limit of the reference range. For females with a uterus, a combined regimen of estrogens and progesterone is recommended until the mean age of natural menopause is reached to reduce the hazards of cardiovascular disease and mortality [1]. Growth hormone supplementation is advisable for enhanced body composition and lipoprotein metabolism, although its clinical benefit on cardiovascular risk remains uncertain. Furthermore, the modality of administration via daily subcutaneous injections is uncomfortable, and adverse effects, such as edema, arthralgia, and myalgia, are commonly reported. Prolactin replacement is currently unavailable [7,13].

Our patient had been living for over 30 years with severe hormonal deficiencies, which affected multiple aspects of her health. She reported impairments in her general energy, physical capacity, temperature regulation, skin characteristics, and sexual life. Moreover, her susceptibility to cardiovascular events and osteoporosis was elevated because of the absence of natural female estrogen protection until menopause. Thyroid and growth hormone deficiencies also contributed to hyperlipidemia, insulin resistance, premature atherosclerosis, and cardiac dysfunction [1]. The adrenal insufficiency posed a significantly high risk for an adrenal crisis during a stressful situation. Fortunately, she had never experienced severe illness nor required a surgical intervention in this period.

## Conclusions

This case report describes a rare clinical syndrome that was left undiagnosed for more than 30 years. A detailed clinical history and physical examination showed several manifestations of hormonal deficiencies, which were subsequently confirmed by laboratory analysis, and cranial imaging revealed the destruction of the pituitary gland. This report aims to highlight the subtle manifestations of Sheehan syndrome to help clinicians promptly diagnose this condition. Even in developed countries such as Portugal, where medical awareness of this disease is insufficient, it is imperative to consider it as a potential cause of hypopituitarism. An early diagnosis can dramatically improve these patients' quality of life and prevent life-threatening conditions arising from severe hormonal deficiencies.
